# Density dependence in *Caenorhabditis* larval starvation

**DOI:** 10.1038/srep02777

**Published:** 2013-09-27

**Authors:** Alexander B. Artyukhin, Frank C. Schroeder, Leon Avery

**Affiliations:** 1 Boyce Thompson Institute and Department of Chemistry and Chemical Biology, Cornell University, Ithaca, NY 14850, USA; 2 Department of Physiology and Biophysics, Virginia Commonwealth University, Richmond, VA 23298, USA

## Abstract

Availability of food is often a limiting factor in nature. Periods of food abundance are followed by times of famine, often in unpredictable patterns. Reliable information about the environment is a critical ingredient of successful survival strategy. One way to improve accuracy is to integrate information communicated by other organisms. To test whether such exchange of information may play a role in determining starvation survival strategies, we studied starvation of L1 larvae in *C. elegans* and other *Caenorhabditis* species. We found that some species in genus *Caenorhabditis*, including *C. elegans*, survive longer when starved at higher densities, while for others survival is independent of the density. The density effect is mediated by chemical signal(s) that worms release during starvation. This starvation survival signal is independent of ascarosides, a class of small molecules widely used in chemical communication of *C. elegans* and other nematodes.

Mechanisms of energy homeostasis and stress response have been a subject of intense research recently. Many important transcription factors, hormones, and signaling molecules, such as FOXO^1^,^2^, insulin^3^,^4^,^5^, TORC^4^,^6^, AMPK^7^,^8^, involved in monitoring and maintaining energy balance are known^9^,^10^,^11^, and new players are being added to this list regularly^12^,^13^. All these master regulators act on intracellular and/or *intra*organismal levels. We know far less about mechanisms acting on the *inter*organismal level to regulate metabolism, energy homeostasis, and stress response. How, if at all, do animals communicate about their energy status? How do they use information obtained from others to maximize their fitness and survival strategy?

Nematodes, *C. elegans* being the most studied representative, lack visual and auditory sensory systems present in higher organisms and therefore have to rely extensively on chemical communication. Since elucidation of the first *C. elegans* dauer pheromone structures^14^,^15^, more than 20 years after its discovery^16^, numerous members of the ascaroside family, derivatives of the dideoxy sugar ascarylose, have been found in *C. elegans* and other nematodes^17^,^18^,^19^,^20^. Ascarosides were originally thought of as a worm waste product, but as we know today, their biosynthesis and release are tightly regulated and reflect the state of the worm^21^,^22^. Even though we are far from understanding the ascaroside language of nematodes in its entirety, almost everything we know today about chemical communication in worms involves ascarosides–dauer formation^22^, mating^23^, attraction^24^, repulsion^25^, aggregation^26^. Ascaroside structures are both highly conserved and diverse across the nematode phylum^18^,^20^,^27^,^28^. Due to this high degree of conservation and apparent importance to worm physiology ascarosides are the most studied class of nematode-specific small molecules.

In its life cycle *C. elegans* goes through four larval stages (L1 to L4) before becoming an adult^29^. When *C. elegans* worms hatch in the absence of food, newly hatched L1 larvae arrest their development and can survive in this state for weeks^11^,^30^. When food becomes available, arrested L1s resume their development and can grow to adulthood. Several signaling networks responsible for monitoring the animal's nutritional status, regulating its metabolic rate, triggering stress response, and governing entry and exit from the L1-arrested state have been discovered^11^. Most of them have conserved functions throughout the animal kingdom, insulin signaling being the most prominent example. Worms with mutated insulin receptor DAF-2 survive starvation twice as long as wild-type animals^31^. At the same time, little is known about communication *between* L1-arrested worms^17^ and whether such communication may help animals to survive starvation. In this work we show that *C. elegans* L1-arrested larvae release chemical cue(s) that make them capable of surviving starvation longer. As a result, worm density has a profound effect on survival rate. Surprisingly, this phenomenon varies a lot among *Caenorhabditis* species and, for example, *C. briggsae* shows a very different behavior. Finally, we present evidence that this signal is not ascaroside-based but is a synergistic blend of several compounds.

## Results

### Some *Caenorhabditis* species survive longer when starved at high density

**Figure 1:**
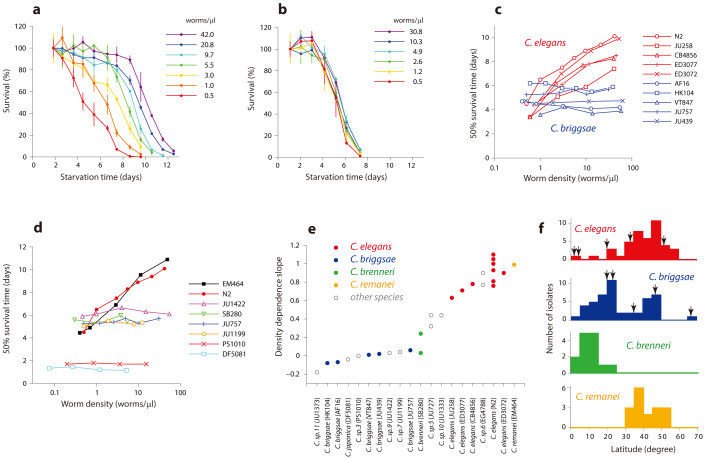
Some *Caenorhabditis* species survive L1 starvation longer when starved at high density. (a) Survival curves of *C. elegans* (N2) in L1 starvation at different worm densities. (b) Survival curves of *C. briggsae* (JU757) in L1 starvation at different worm densities. (c) Effect of worm density during starvation on 50% survival time for various wild isolates of *C. elegans* and *C. briggsae*. Slopes of the curves reflect the magnitude of the density dependence. (d) Effect of worm density during L1 starvation on 50% survival time for several representative *Caenorhabditis* species. (e) Magnitude of the density effect in L1 starvation for all tested *Caenorhabditis* species, including various strains of *C. elegans* and *C. briggsae*. Density dependence slopes were obtained from linear regressions of 50% survival time as a function of log_2_(worm density). (f) Latitude distributions of four *Caenorhabditis* species constructed based on data from^54^. Arrows in *C. elegans* and *C. briggsae* distributions indicate locations where worms tested in this work (Fig. 1c) had been isolated. Note log scale for *x*-axes in (c) and (d).

While studying L1 starvation of *C. elegans* (N2), we found that L1-arrested worms survived starvation in buffer longer when they were starved at higher worm density (Fig. 1a). The effect was significant: 50% survival time doubled with a 100-fold increase in worm density from 0.5 to 50 worms/μl. We also noticed that in extended starvation worms that were starved at higher density developed faster and overall looked healthier during post-starvation recovery. We observed a similar density effect on L1 starvation on plates. However, since it is hard to perform this kind of experiment quantitatively on plates, all results that we present in this paper were obtained for worms suspended in M9 buffer. The dependence of survival on worm density leveled off at both low and high densities and the strongest effect was between 1 and 10 worms/μl. A similar experiment with *C. briggsae* yielded a surprising result–worm density had no effect on survival of *C. briggsae* L1 larvae (Fig. 1b). To verify that these observations were not artifacts inherent to lab strains (N2 had been in continuous lab culture for many years before being frozen and has acquired mutations and phenotypes absent in wild *C. elegans*), we repeated the experiments with several wild isolates of *C. elegans* and *C. briggsae*. The results were consistent with our first observations–all *C. elegans* strains that we tested had a positive effect of worm density on survival, whereas all *C. briggsae* strains showed no density effect (Fig. 1c). We then examined several other *Caenorhabditis* species (12 of 26 known at the time) to check how general the density effect in L1 starvation was. Nine species did not have the density dependence, but 3 out of 12, including *C. elegans*, did (Fig. 1d, e). There was no obvious correlation between the density dependence and position in the phylogenic tree or mode of reproduction (hermaphrodite vs male/female species) (Fig. S1). The only correlation that we could notice was with geographic distribution of the species, namely the latitude range that the species occupies (Fig. 1e, f). For species preferring higher latitudes, such as *C. elegans* and *C. remanei*, L1 survival depended on the worm density, while for species living in warmer climates, such as *C. briggsae* and *C. brenneri*, it did not. However, within a species, the location from which a strain had been isolated did not affect its density dependence profile (Fig. 1c, f). Information about the habitats and distribution of many *Caenorhabditis* species is spotty and it is hard to say at this point if the correlation between the density dependence and geographic distribution is meaningful.

### The density effect is mediated by chemical cue(s)

**Figure 2:**
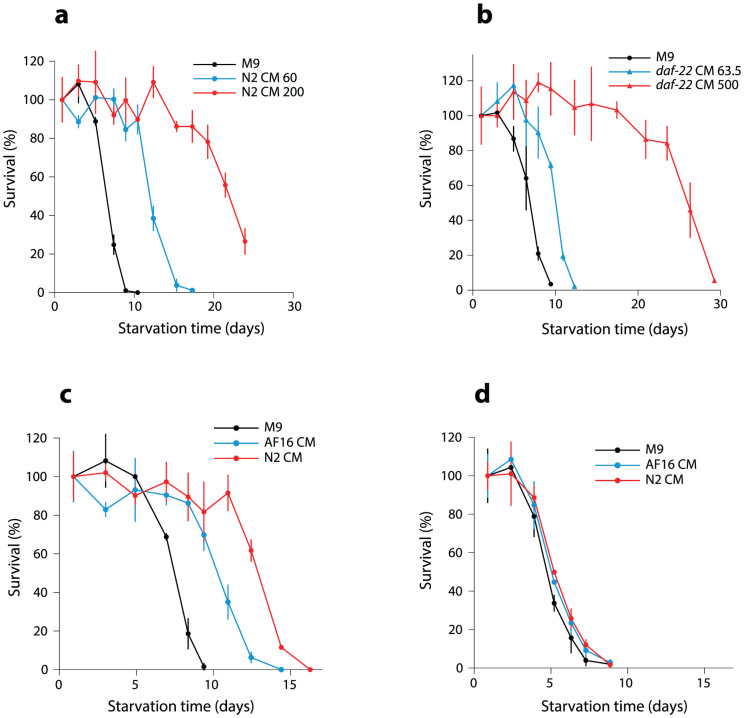
Conditioned medium (CM) from high density L1s extends survival of N2 worms at low density. (a) CM from high density N2 L1 larvae extend survival of N2 L1s at low density (0.8 worms/μl) in a dose dependent manner. Shown in black is the survival curve in M9 buffer. Numbers in the legends of (a) and (b) correspond to density of worms during conditioning (worms/μl). (b) CM from *daf-22* L1 worms has survival extension effects similar to those of N2 CM. (c) CM from *C. briggsae* (AF16) L1s also extends survival of *C. elegans* (N2) in L1 starvation, though the effect appears to be smaller compared to N2 CM. (d) In contrast, neither *C. elegans* (N2) nor *C. briggsae* (AF16) CMs has an effect on L1 survival of *C. briggsae* (AF16) at moderate CM densities. For (c) and (d) CM was prepared at 60 worms/μl. CMs collected at 10-fold higher densities extend survival of both *C. elegans* and *C. briggsae* (Fig. S15).

Worm density may exhibit its effect through either mechanical or chemical interactions. Both have been shown to influence *C. elegans* development and life traits^32^,^33^,^34^. To check whether L1 density dependence is mediated by chemical cues, we collected the medium from high density L1-arrested worms and studied its effect on starvation survival of L1 larvae at low density. Conditioned medium (CM) extended L1 survival (Fig. 2a) indicating that the density effect is mediated at least in part by released chemical(s). Consistent with this observation, presence of polymer beads during starvation, mimicking purely mechanical interactions, had no positive effect on survival (Fig. S2). Several simple tests with cut-off filters (Fig. S3a) and lyophilization (Fig. S3b) of CM led us to conclude that the active component was nonvolatile small molecule(s) (<3 kDa), suggesting that it might be ascaroside(s). Arrested L1 *C. elegans* worms produce and release a blend of about 20 ascarosides (Fig. S4), some of them being specific to the L1 stage^17^. *daf-22* is a standard negative control in experiments on ascaroside bioactivity^35^,^36^. *daf-22* encodes the peroxisomal 3-keto-acyl-CoA thiolase, which is necessary for peroxisomal β-oxidation of fatty acids and ascaroside biosynthesis^35^,^37^,^38^. Thus, null mutants of *daf-22* do not produce any short-chain ascarosides (Fig. S4). We tested the effect of *daf-22* CM in L1 survival of N2 worms and found that it had an effect similar to wild-type CM (Fig. 2b). This result rules out ascarosides as possible active ingredients of CM.

We then studied survival of *C. elegans* L1 larvae in CM from *C. briggsae.* Although *C. briggsae* did not have the density effect, *C. briggsae* CM extended L1 survival of N2 worms (Fig. 2c). Combined with the lack of an effect in the opposite experiment, i.e. *C. elegans* CM on *C. briggsae* L1 survival (Fig. 2d), this result suggests that *C. briggsae*, and probably other *C.* species which do not have the density dependence, produce the signal responsible for longer survival at higher densities but do not respond to it. To test this hypothesis we studied the effect of CM from 2 other species on N2 L1 survival and found that they also had a positive effect (Fig. S5). Taken together, these results demonstrate that the density effect in L1 starvation is quite general and is mediated by small molecule(s) released by starved worms of various species. For reasons that we do not currently understand some species are able to survive starvation longer in response to this signal while others are not.

### The density effect requires chemosensory neurons

**Figure 3:**
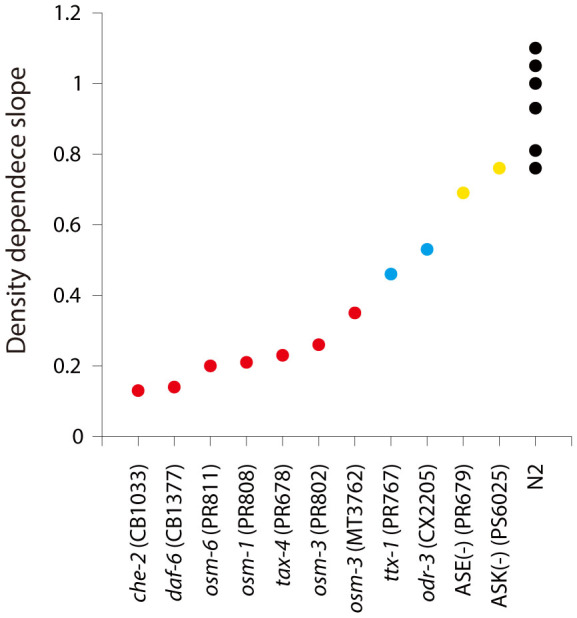
*C. elegans* chemosensory mutants have diminished density dependence compared to wild-type. Magnitude of the density effect on L1 starvation for mutants with impaired sensing of soluble chemicals (red), thermosensory and olfactory mutants (blue), worms engineered to lack ASE or ASK chemosensory neurons (yellow), and wild type (black).

Since the density effect involves small-molecule signals, we hypothesized that the response pathway may start with sensory neurons, where these signals are first detected. In keeping with this model, we found that several chemosensory mutants had no or greatly diminished density dependence (Fig. 3). In contrast, a thermosensory mutant (*ttx-1*) and a mutant with impaired sensing of volatile chemicals (*odr-3*) had intermediate density dependence. We then tested if two individual neurons types, ASE and ASK, are required for the density effect. ASE neurons are required for normal chemotaxis to several soluble chemicals^39^, while ASK expresses receptors for ascr#2, one of the dauer pheromones^40^,^41^, and is involved in social behavior^41^,^42^. Worms genetically engineered or mutated to lack ASK^26^ or ASE^43^ sensory neurons had density dependence similar to wild-type, indicating that neither of these two neuron types is necessary for the density effect (Fig. 3). However, we have not verified ablation of these neurons in L1 stage in these strains.

### The density effect is not due to nutrients released during hatching

**Figure 4:**
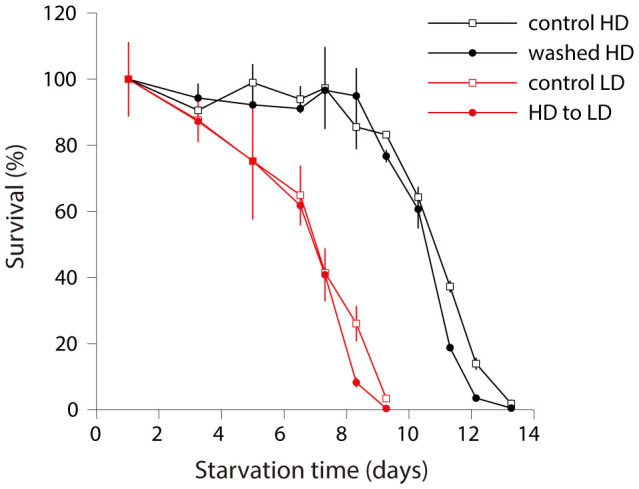
Active component is released after hatching and is necessary past 24 hours to affect survival. N2 L1 worms were starved continuously at high density in the same medium (control HD), at high density but with a wash and fresh buffer after the first 24 hours of starvation (washed HD), continuously at low density (control LD), at high density for the first 24 hours and then diluted to low density (HD to LD).

What is the active component of CM responsible for extending survival? The experiment with *daf-22* CM already excluded ascarosides. The density effect is not limited to interaction between conspecifics as we showed that *C. elegans* L1 larvae respond to signal(s) produced by other species, though we do not know whether different species produce the same signal. The simplest possibility for a very general, nonspecific cue is the nutrients released during hatching. To explore this possibility we need to answer several questions. First, when is the active component released? Is it a one-time pulse at the moment of hatching or is it continuously released by starved worms? In the latter case the active component is less likely to be a nutrient. Second, can nutritional energy sources extend L1 survival? A recent report suggests that they may^44^. If so, does CM have those nutrients in sufficient amounts to account for the observed effect in survival? To address the first question, we performed a density shift experiment where we compared control worms starved at high density, worms starved at high density which were washed after the first 24 h of starvation, and control worms starved at low density (Fig. 4). The wash after 24 h had no effect; the high density control and high density washed worms were indistinguishable. To check for the possibility that a short exposure to the active component in the beginning of starvation is sufficient to account for long term survival benefits, we included a control where worms hatched at high density were diluted to low density after 24 h. These worms had the same survival as control low density worms (Fig. 4). From these results we conclude that the active component of CM responsible for the density effect is released beyond hatching and the first day of starvation and L1 worms have to be exposed to it for more than a day to be able to survive longer.

To answer the second question, we studied L1 starvation survival in the presence of various nutrients. All nutrients that we tested (bacteria, D-glucose, amino acid mixture, BSA, acetate, ethanol) extended L1 starvation survival of N2 worms but only at relatively high concentrations (Fig. S6–S10). In all cases concentrations necessary for the effect were in mM range or greater. L-glucose, an unnatural enantiomer of D-glucose with no nutritional value, had no effect (Fig. S7b). The lack of an L-glucose effect, together with the observation that ethanol had no effect on *sodh-1* mutants (data not shown; *sodh-1* encodes alcohol dehydrogenase necessary for the first step of ethanol metabolism^45^), indicate that nutrients have to be metabolized and used as energy sources to have a positive effect on starvation survival (see also^44^). To determine whether nutrients are present in CM in such high concentrations, we profiled the composition of CM by LC-MS and 2D NMR. CM had a complex composition and contained amino acids, nucleosides and their degradation products, ascarosides, lysolipids, fatty acids, ceramides and other metabolites (data not shown). However, concentrations of even the most abundant molecules were in the μM range, and for CM from 100 worms/μl did not exceed 100 μM. This is at least an order of magnitude lower than concentrations necessary for nutrients to affect starvation survival. We also noticed that in the experiments with nutrients survival curves changed their slope and became progressively less steep with increasing nutrient concentration (Fig. S6–S10), indicating higher heterogeneity of starving worm populations in the presence of nutrients. On the other hand, survival curves in CM experiments had the same slopes as in M9 but were parallel-shifted to longer times (Fig. 2). This difference suggests that mechanisms by which CM and nutrients extend L1 starvation survival are not the same. Finally, *C. briggsae* worms and *C. elegans* chemosensory mutants, which showed no or little density dependence, survived longer when supplied with a nutrient (D-glucose, Fig. S11–S13). Taken together, all these lines of evidence indicate that the survival extension effect of CM is different from simply providing nutrients to starving worms.

### The density effect is mediated by a synergistic blend of chemical cues

**Figure 5:**
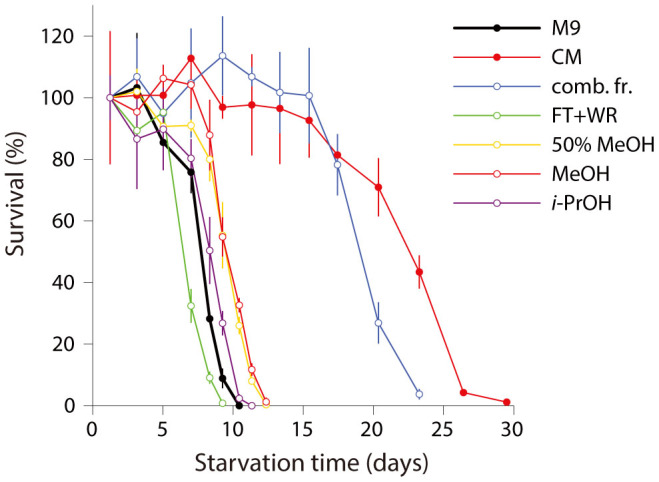
CM fractions have a synergistic effect on starvation survival. Survival curves of N2 L1s in M9, unfractionated CM, four individual CM fractions (flow-through and water rinse (FT + WR), 50% methanol, methanol, propanol-2), and a combination of all four fractions. The four fractions had no or little effect on starvation survival individually but had a strong positive effect similar to that of unfractionated CM when applied in combination.

In an attempt to isolate the active component of CM we performed activity guided fractionation using reverse phase solid phase extraction. We consistently found that while the combination of all fractions was active, individual fractions had no or little activity (Fig. 5), pointing to a synergistic effect among several components. This result is not surprising as most natural pheromones are mixtures of more than one ingredient that often act in synergy^46^. The synergistic effect complicates isolation of the CM active component(s). We continue working in this direction.

## Discussion

Ascaroside signaling is implicated in important behaviors and developmental decision points of the worms. Modular structures with the conserved ascarylose core facilitate ascaroside characterization and synthesis and contribute to their popularity among nematode chemical biologists. Far less, if anything, is known about other molecules used in nematode communication. Although there is evidence in the literature for their existence^35^,^47^,^48^, no molecules have been isolated and characterized. Here, we present an example of ascaroside-independent communication among *C. elegans* individuals. The density dependence of L1 starvation survival, resembling quorum sensing in bacteria, shows that worms can signal to each other in starvation. Apparently, this signaling enables the worms to adjust their metabolism and/or stress response and ultimately survive longer. The most intriguing question (besides the identity of the signaling molecule(s) and pathway(s) activated in worms, of course) is why this response is different even among the species of the same genus. Why do some species survive longer when starved in dense groups while others do not? We are only now beginning to learn about *Caenorhabditis* ecology and have yet to link field studies with laboratory observations. We speculate that the correlation of the L1 density dependence with latitude, and consequently climate, may be related to survival through cold winters, since the L1 is the stage in the worm life cycle that best survives freezing^49^. We also have preliminary evidence that goes beyond L1 starvation that *C. elegans* are in general more communal than *C. briggsae*, but this remains to be further explored.

## Methods

### Worm strains

We used the following wild strains in this study. *C. elegans* N2, CB4856, JU258, ED3072, ED3077; *C. briggsae* AF16, HK104, VT847, JU757, JU439; *C. remanei* EM464; *C. brenneri* SB280; *C. japonica* DF5081; *C. sp. 3* PS1010; *C. sp. 5* JU727; *C. sp. 6* EG4788; *C. sp. 7* JU1199, *C. sp. 9* JU1422; *C. sp. 10* JU1333; C. sp. 11 JU1373. We used the following *C. elegans* mutant strains. DR476 *daf-22*, CB1033 *che-2*, CB1377 *daf-6*, PR811 *osm-6*, PR808 *osm-1*, PR678 *tax-4*, PR802 *osm-3*, MT3762 *osm-3*, PR767 *ttx-1,* CX2205 *odr-3*, PR679 *che-1*, PS6025 qrIs2 [sra-9::mCasp1]. Some strains used in this study were obtained from Caenorhabditis Genetics Center.

### L1 survival assay

We maintained worms at 20°C on nematode growth medium (NGM) plates seeded with *E. coli* HB101. We transferred 2–4 gravid adults to new plates before food was exhausted (every 3 days) to make sure that worms did not experience starvation for at least 4 generations prior to the experiment. We washed a mixed population of worms from 2–3 6 cm plates and treated them with alkaline bleach in a plastic 15 ml tube (8 ml H_2_O + 2 ml bleach + 0.3 ml 10 M NaOH) for 6 min with intermittent vortexing. This treatment yielded sterile egg suspension. After 2 washes with M9 buffer (10 ml each) we resuspended eggs in 3 ml M9 and let them hatch and arrest at L1 stage for synchronization (20°C). 18 to 27 h after the egg prep we seeded L1 larvae on six 10 cm NGM plates with HB101 lawn at 1500–1800 L1s/plate. 60 to 66 h later (at 20°C), when bacterial food was still present but there were many eggs on the plates, we washed worms and eggs off the plates and bleached them as described above. After 2 M9 washes and resuspension in 3 ml M9 we obtained egg suspension with ca. 30–50 eggs/μl, which was diluted with M9 to desired final density.

This procedure is typical for N2, other strains and *C.* species required some adjustments, e.g. more plates to start with to get enough eggs or longer bleaching times. In all cases we made sure that resulting egg suspension was clean and did not contained undissolved worm bodies or fragments. For starvation experiments we used either BSA-coated 15 ml plastic tubes (tubes were filled with 0.1% BSA for 1–3 h and washed 3 times with water. It is important to maintain sterility during this process) or 40 ml glass vials (glassware was cleaned by soaking in concentrated NaOH overnight, thoroughly rinsed with water and sterilized by autoclaving). For experiments presented in Fig. 1 and 3 we used 3 ml of egg suspension in BSA-coated plastic tubes, for experiments presented in Fig. 2, 4, and 5 we used 6 ml of egg suspension in glass vials. Starvation experiments shown in Fig. 2 and 5 we performed at low worm density (0.5–1 worm/μl), for others the final worm density during starvation was variable, as indicated on the graphs. All starvation experiments were done at 25°C. This temperature is the upper end of *C. elegans* comfort range^50^. *C. briggsae*, on the other hand, can tolerate higher temperatures^51^,^52^. One may question if the density dependence that we report in this paper is related to heat stress. We observed similar density effects in *C. elegans* L1 starvation at lower temperatures (20–22°C, data not shown), and no density effect in *C. briggsae* at 34°C, where it is most likely to be heat shocked (Fig. S14). Therefore, we believe that the density dependence is not due to heat stress.

Starvation cultures were mixed by rocking (plastic tubes) or shaking (at 210 rpm, glass vials). To monitor starvation survival we took triplicate aliquots from the samples every day or every other day and plated them on NGM plates with HB101 for recovery. After 2 days of recovery at 20°C we counted and removed alive worms that resumed growth and developed past L1 stage. We inspected the plates after additional 24 h to count worms that developed slower and were missed in the first round of counting. For the first 5 days of starvation (N2) the extra day of recovery did not affect the numbers significantly (counts on the 3^rd^ day were less than 5% of the counts for the first 2 days of recovery). However, after longer starvations worms recovered progressively slower and more heterogeneously (see also^53^), so it was important to remove fast recovering worms after 2 days and count slower growing worms after another day or two. Survival curves presented on the same graph are for samples from the same stock egg suspension which were starved in parallel. Error bars represent standard deviation from triplicate recovery plates for each time point.

### Preparation of L1 conditioned medium (CM)

To obtain CM from L1s arrested at high density we grew worms in liquid culture, which allows to scale up egg production by 10- to 100-fold compared to worms grown on plates. Liquid cultures were started with synchronized L1 larvae, obtained as described above and grown at 22°C, 220 rpm in S-complete medium supplemented with 1–2% (w/w) *E. coli* HB101 or K12. For a small scale liquid culture we inoculated 25 ml S-complete in a 250 ml flask with 7·10^4^ synchronized L1s and added 1 ml 50% *E. coli* stock suspension. We monitored the worm culture during the next 2 days and added *E. coli* as it became depleted. Bleaching after 52–56 h growth yielded ca. 10^6^ eggs (100 μl egg pellet), which, when resuspended in 20 ml M9, gave 50 L1s/μl suspension after hatching. After 24 h shaking at 22°C in a 125 ml flask, spinning L1s, and filtering the supernatant through a 1 μm glass fiber syringe filter, we obtained the CM50 (the number indicates worm density during conditioning). For a large scale liquid culture we inoculated 250 ml S-complete in a 2 l flask with 7·10^5^ synchronized L1s obtained from a small scale liquid culture and added 10 ml 50% *E. coli* stock suspension. We monitored the worm culture during the next 2.5 days and added *E. coli* as it became depleted. Bleaching after 58–62 h growth yielded ca. 10^7^ eggs (1 ml egg pellet), which, when resuspended in 20 ml M9, gave 500 L1s/μl suspension after hatching. After 24 h shaking at 22°C in a 125 ml flask, spinning L1s, and filtering the supernatant through a 1 μm glass fiber syringe filter, we obtained the CM500.

### HPLC-MS analysis of CM

HPLC-MS and HPLC-MS/MS was performed using an Agilent 1100 Series HPLC system equipped with an Agilent Eclipse XDB-C18 column (4.6 mm × 250 mm, 5 μm particle diameter) connected to a Quattro II mass spectrometer (Micromass/Waters) using a 1:1 split. For HPLC, a 0.1% aqueous acetic acid-acetonitrile solvent gradient was used at a flow rate of 1 ml/min, starting with an acetonitrile content of 5% for 5 min which was increased to 100% over a period of 40 min. Samples were analyzed by HPLC-ESI-MS using a capillary voltage of 3.5 kV in negative ion mode and a cone voltage of −40 V or −20 V or positive ion mode and cone voltage of +30 V. HPLC-MS/MS screening for precursor ions of *m*/*z* 73 was performed using argon as collision gas at 2.1 mtorr and 30 eV. CM was either analyzed directly without further processing or frozen, lyophilized, extracted with methanol, and then analyzed by HPLC-MS or MS/MS.

### Activity guided fractionation

We used reverse phase solid phase extraction to fractionate CM. 12 ml of CM500 from a large scale liquid culture was loaded on an Agilent Mega Bond Elute C18 cartridge (60 ml cartridge, 10 g sorbent) prewashed with propanol-2 and methanol (50 ml each) and equilibrated with 50 ml water. We then rinsed the column with water, 50% methanol, methanol, and propanol-2 (50 ml each) and collected four fractions: flow through + water rinse (FT + WR), 50% methanol, methanol, and propanol-2. We took a half of each fraction and mixed them to create a combined fraction. Since volume of each starvation sample is 6 ml, 12 ml of original CM is sufficient for 2 samples – one with individual fractions and one with the combined fraction. We also saved 6 ml of unfractionated CM, which was tested in the same experiment (Fig. 5). We froze the FT + WR fraction (corresponding to 6 ml of CM), lyophilized it, and reconstituted in 6 ml H_2_O to get the original buffer concentration. We evaporated solvent from the three organic fractions (in vacuum at RT), redissolved them in 60 μl methanol, and mixed with 6 ml M9 each. We verified that 1% methanol in starvation samples did not affect L1 survival. We evaporated organic solvents from the combined fraction, froze the aqueous residue, lyophilized it, and reconstituted with 6 ml H_2_O. Since handling of fractions is done in nonsterile conditions, it is easy to get them contaminated with bacteria after removal of organic solvent, which might affect the outcome of starvation experiments. In later experiments we filtered all samples through prewashed 0.2 μm syringe filters immediately prior to adding eggs. We found that this filtration did not affect activity of CM.

### Statistical analysis

We performed unpaired two-tailed t-tests comparing density dependence slopes of different *Caenorhabditis* species with zero and comparing density dependence slopes of *C. elegans* (N2) with various wild isolates of *C. briggsae*. In the latter case t-statistic was calculated according to the following formula 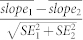
where *SE_i_* is the standard error of *slope_i_*. p-values were obtained using (N − 4) degrees of freedom, where N = n_1_ + n_2_ is the number of data points for samples 1 and 2. For experiments with conditioned medium and its fractions we performed t-tests comparing 50% survival times at different conditions. 50% survival times (*t_half_*) were obtained from fitting survival data with a sigmoid function 
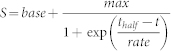
where *base*, *max*, *t_half_*, and *rate* are fitting parameters. *base* and *max* are close to 100 and −100, respectively, and may be held constant during fitting. Readers particularly interested in results of the statistical analysis can find them in Table S1.

## Author Contributions

A.A. conceived the study, designed and performed experiments, analyzed the data, drafted and revised the manuscript. F.S. participated in the design of experiments and data analysis and provided materials and equipment. L.A. conceived and coordinated the study and designed experiments. All authors read and approved the final manuscript.

## Supplementary Material

Supplementary Information Supporting info
