# Supplementary material for: Density dependence in Caenorhabditis larval starvation
Source: Sci Rep. 2013 Sep 27;3:2777. doi: 10.1038/srep02777 (PMC3784960; doi:10.1038/srep02777)
Supplement: Supplementary Information — Supporting info [file srep02777-s1.pdf]

Supporting Information for

Density dependence in *Caenorhabditis* larval starvation

Alexander B. Artyukhin, Frank C. Schroeder, and Leon Avery

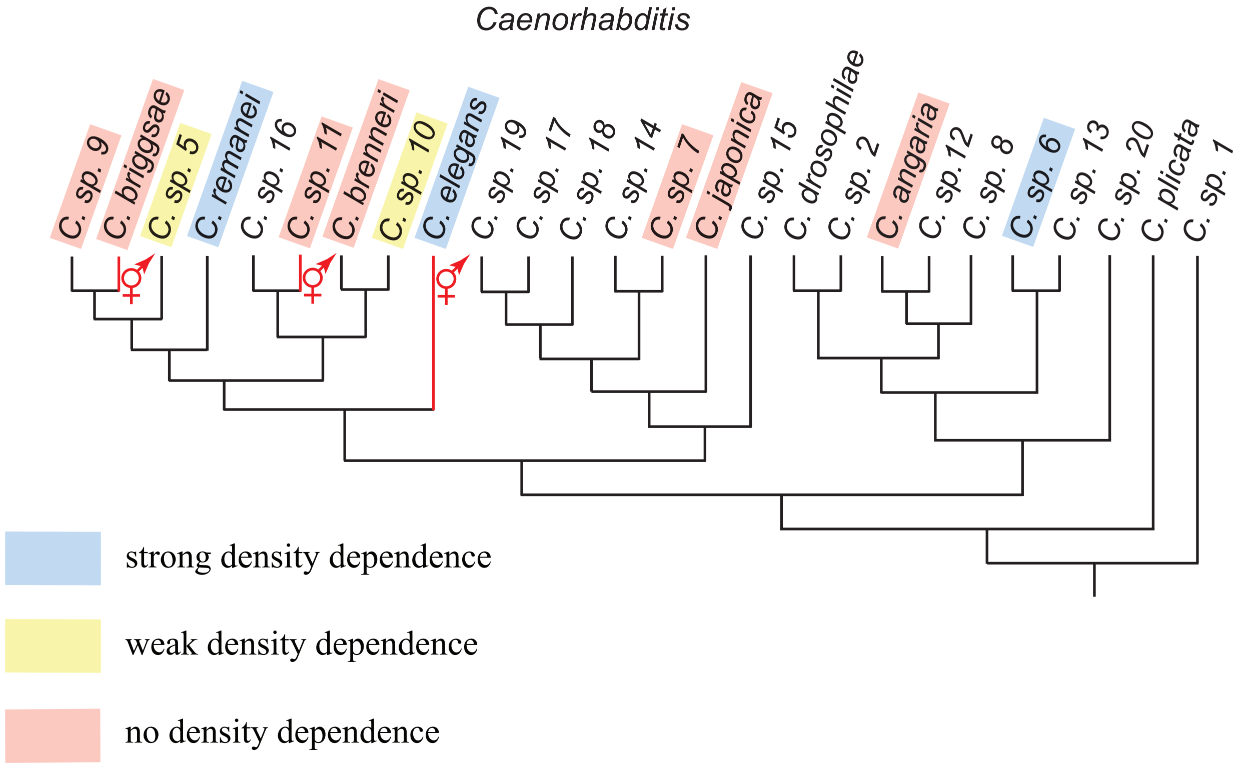

Figure S1. Phylogeny of *Caenorhabditis* species (based on Ref. S1) showing no correlation between density dependence and proximity in phylogeny or mode of reproduction.

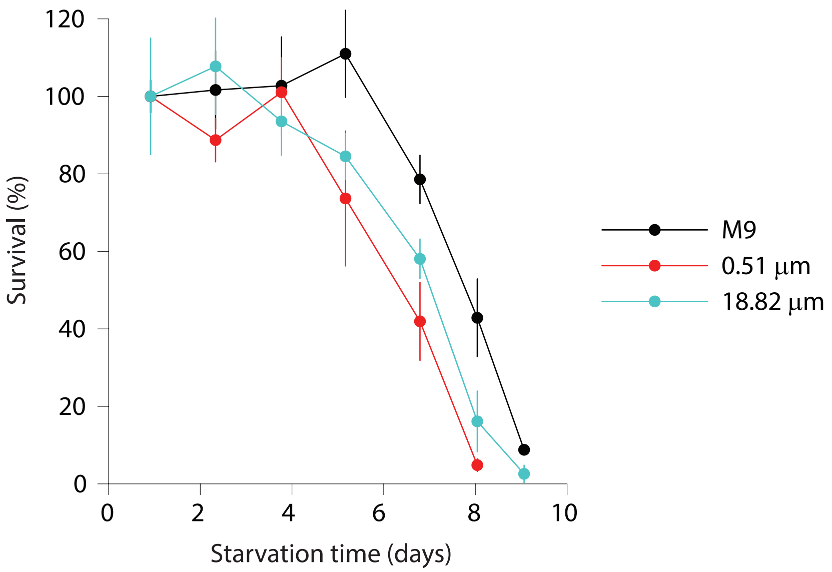

Figure S2. No positive effect of mechanical interactions in L1 starvation. N2 worms were starved in M9 buffer alone or in the presence of carboxylated polystyrene beads of two sizes, 0.51 μm (final density 0.2 weight % or $2.8 \cdot 10^7$ beads/μl) and 18.82 μm (final density 0.2 weight % or $5.4 \cdot 10^2$ beads/μl).

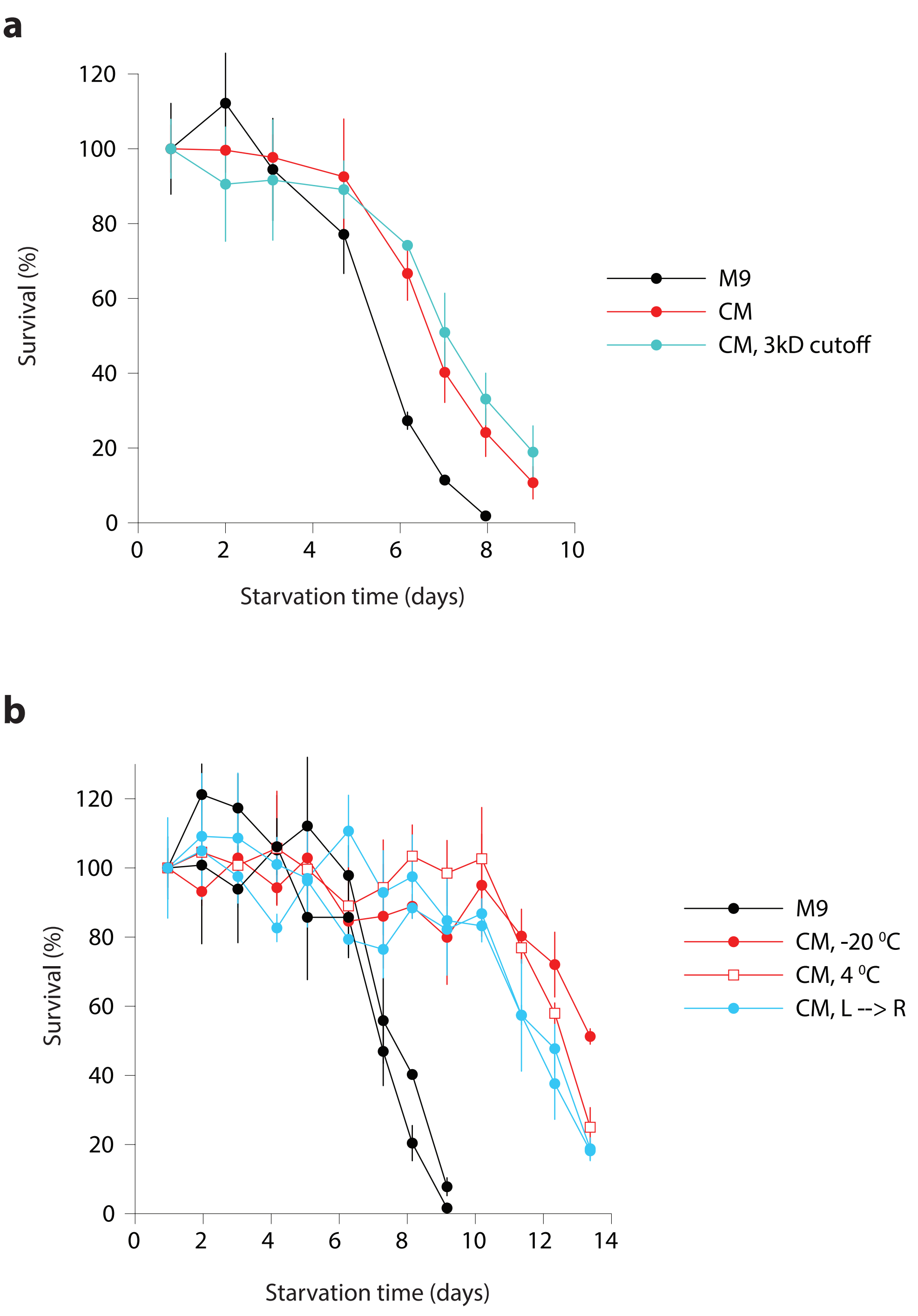

Figure S3. Treatments of CM. (a) Passing through 3 kD cut off filter did not affect the ability of CM50 to extend survival. (b) Lyophilization and reconstitution in water did not significantly affect the ability of CM50 to extend survival. Prior to the starvation experiment CM was stored at either 4 ^{0}C or -20 ^{0}C, which did not affect its activity. Duplicate curves for the M9 control and lyophilized/reconstituted CM are for two starvation samples run in parallel and prepared from the same batch of eggs.

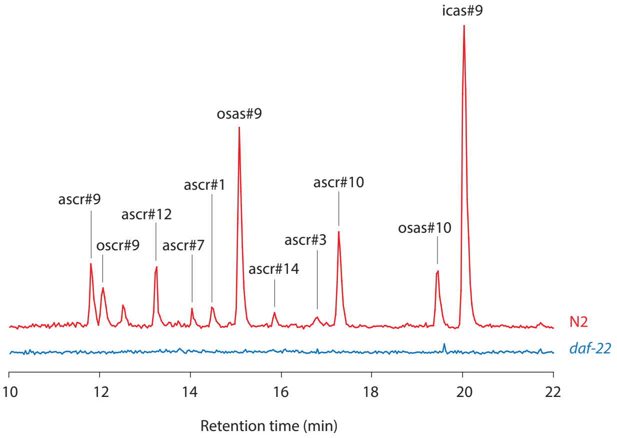

Figure S4. Total ion current (TIC) chromatograms of LC-MS/MS scans for parents of m/z 73 (Ref. S2) showing ascaroside profiles of MeOH extracts of N2 and *daf-22* L1 medium. Some ascarosides present in the N2 sample are not visible in the TIC either because they do not produce m/z 73 fragment (ascarosides without carboxy terminal groups, such as ascr#2, ascr#4, ascr#6, osas#2), or they are masked by other, more abundant ascarosides (tsas#9), or they are present in small quantities (ascr#18, tsas#10). We verified that neither of these ascarosides is present in the *daf-22* sample. Ascarosides are named according to smid-db.org.

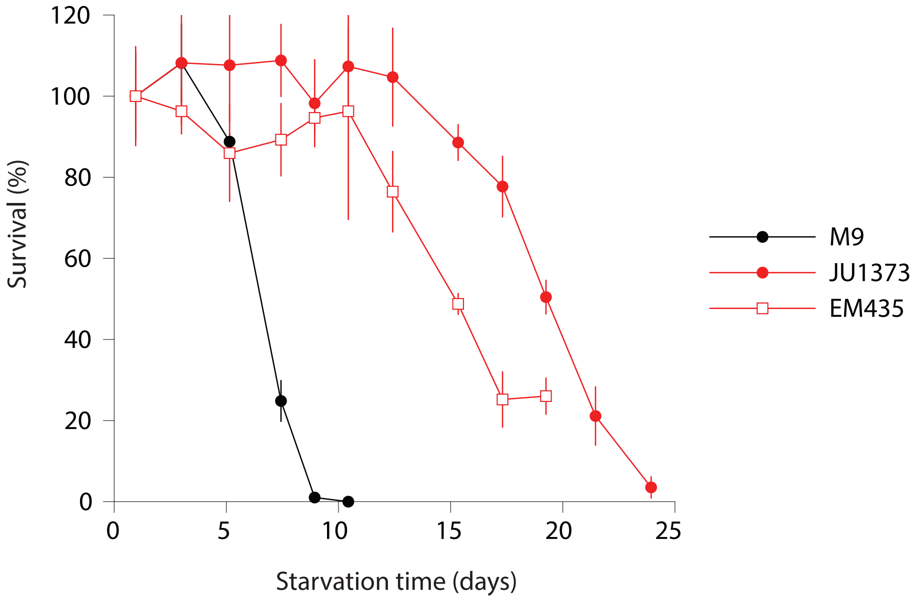

Figure S5. CM500 from *Caenorhabditis sp. 11* (JU1373) and CM250 from *Oscheius myriophila* (EM435) extended N2 L1 starvation survival.

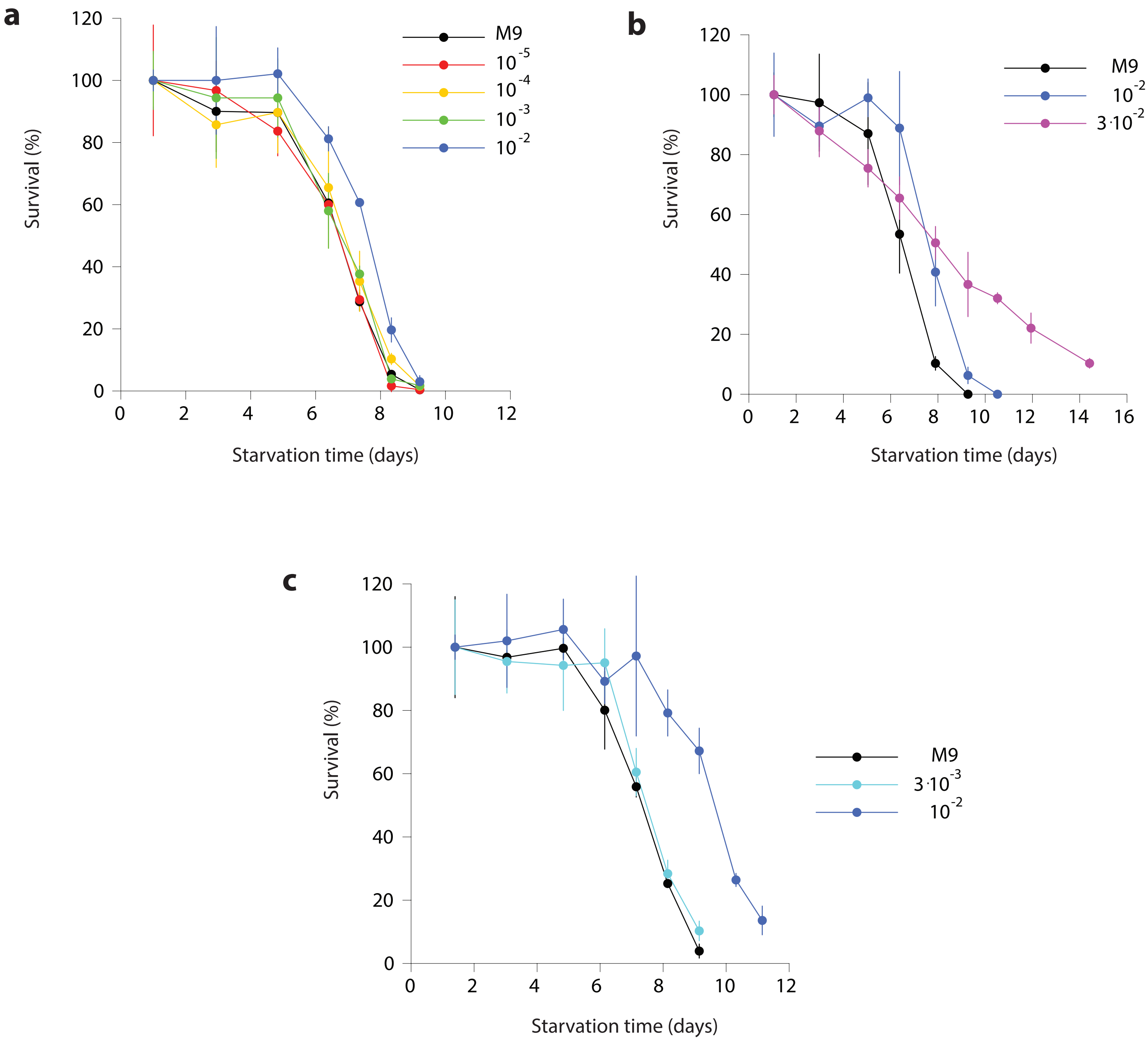

Figure S6. Effect of bacterial food (HB101) on N2 L1 starvation survival. Legend numbers indicate bacterial concentrations relative to the typical concentration used to feed worms in liquid cultures, which is 3% (w/w). (a) Low concentrations of bacteria (10^{-5} to 10^{-3}) had no effect on L1 starvation. (b) Starved L1s survived longer at intermediate bacterial concentrations (10^{-2} and $3{\cdot}10^{-2}$). At higher concentrations worms exited L1 arrest and developed to become dauers (10^{-1}) or adults ($3{\cdot}10^{-1}$). For experiments shown in (a) and (b) we used frozen HB101 stock. (c) For this experiment we used freshly grown bacteria. Starved L1s survived longer at intermediate bacterial concentration (10^{-2}) but the lower concentration $3{\cdot}10^{-3}$ had no effect. At higher bacterial concentrations worms exited L1 arrest and developed to become dauers ($3{\cdot}10^{-2}$) or adults (10^{-1}).

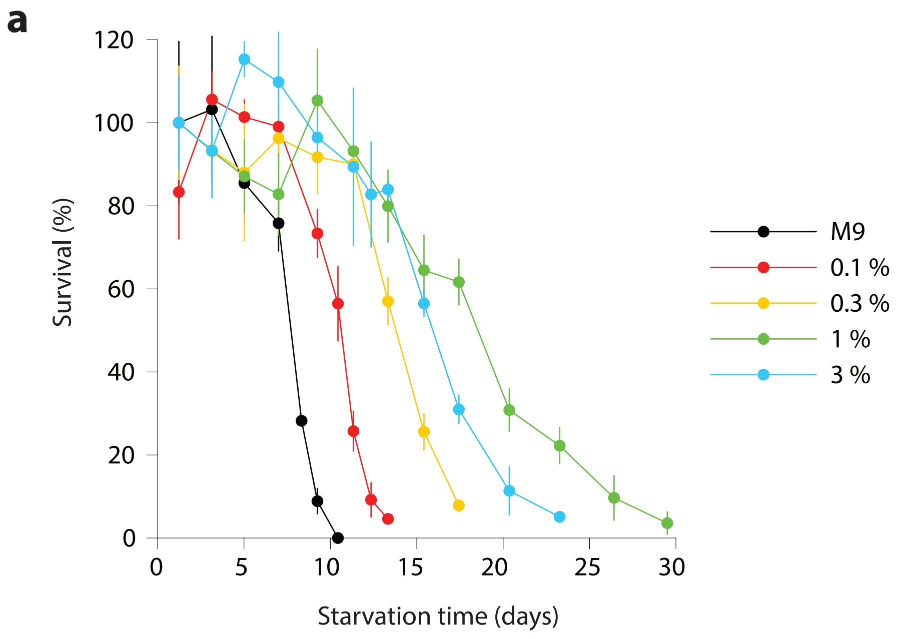

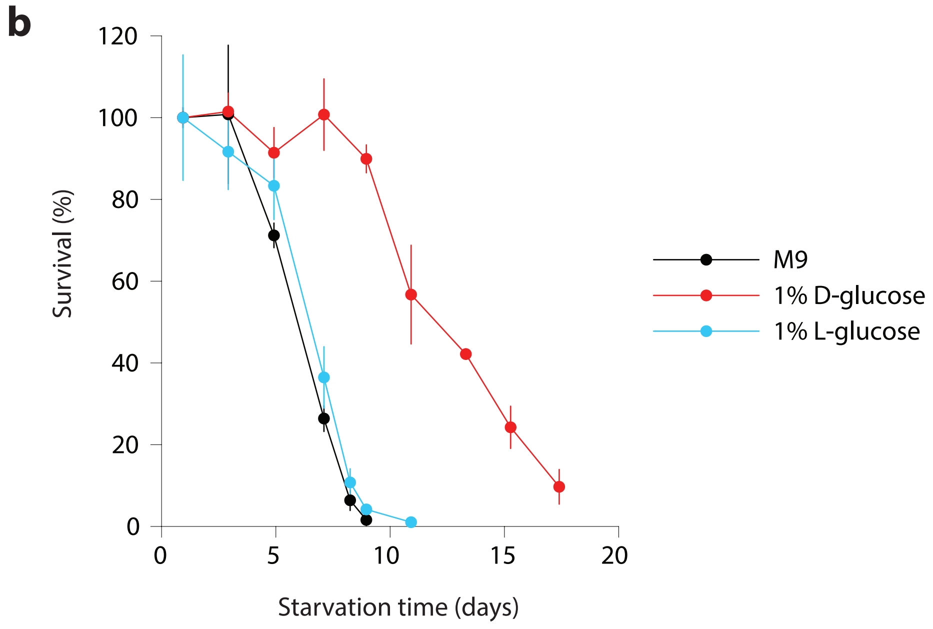

Figure S7. Effect of glucose on N2 L1 starvation survival. (a) D-glucose extended L1 survival. Legend numbers indicate glucose concentration (w/w). 1% is equal to 56 mM. (b) L-glucose had no effect.

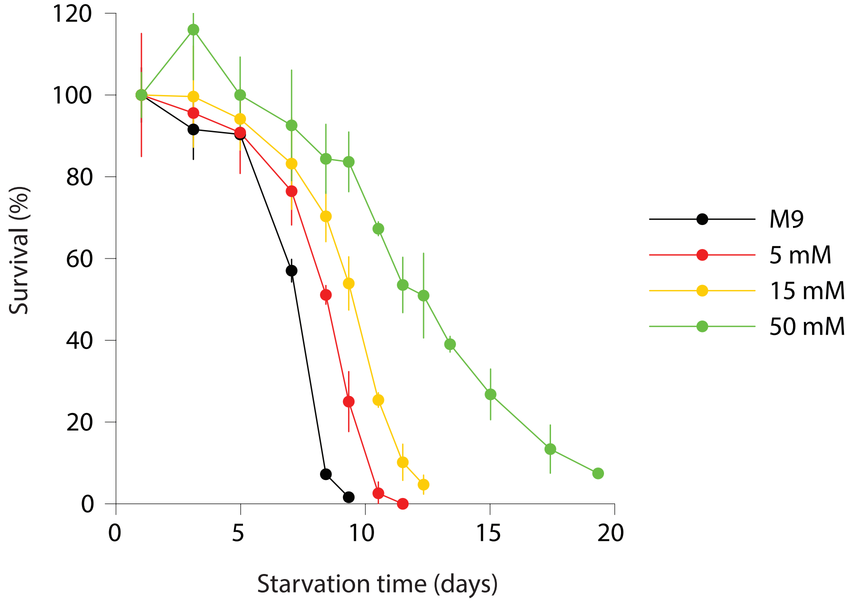

Figure S8. Effect of potassium acetate on N2 L1 starvation survival.

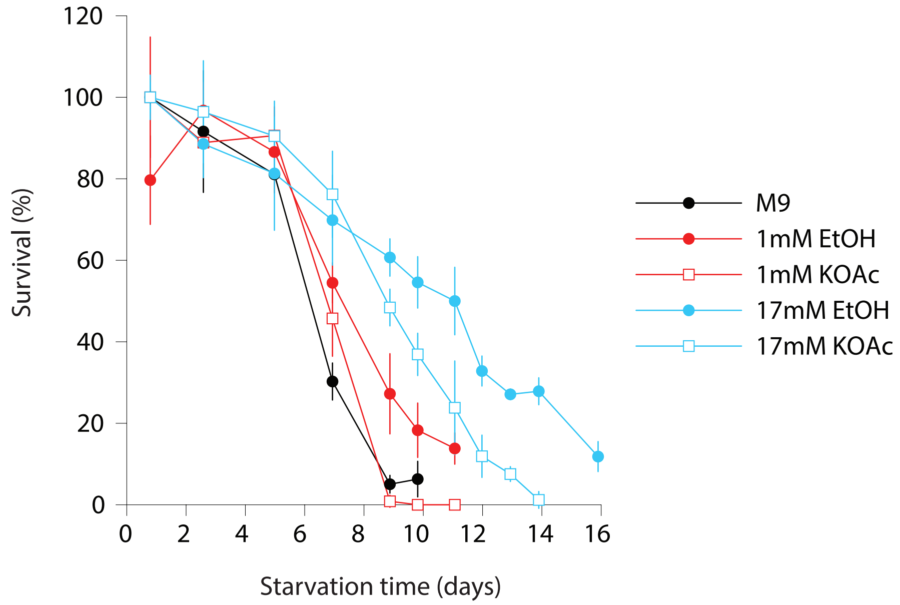

Figure S9. Effect of ethanol and potassium acetate on N2 L1 starvation survival.

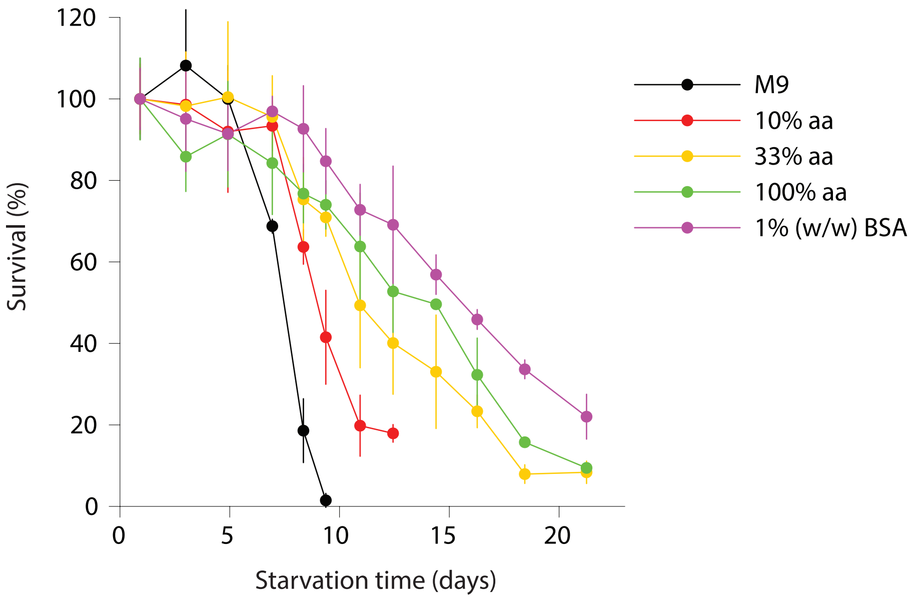

Figure S10. Effect of amino acid mixture and bovine serum albumin (BSA) on N2 L1 starvation survival. Amino acid mixture contained 19 amino acids according to the recipe of chemically defined medium for axenic cultivation of *C. elegans*[S3]. 100% aa refers to this mixture (total concentration of amino acids 120 mM or 1.5 % by weight) without dilution. 33% aa and 10% aa are 3- and 10-fold dilutions of this mixture, respectively. BSA concentration is 1 % by weight.

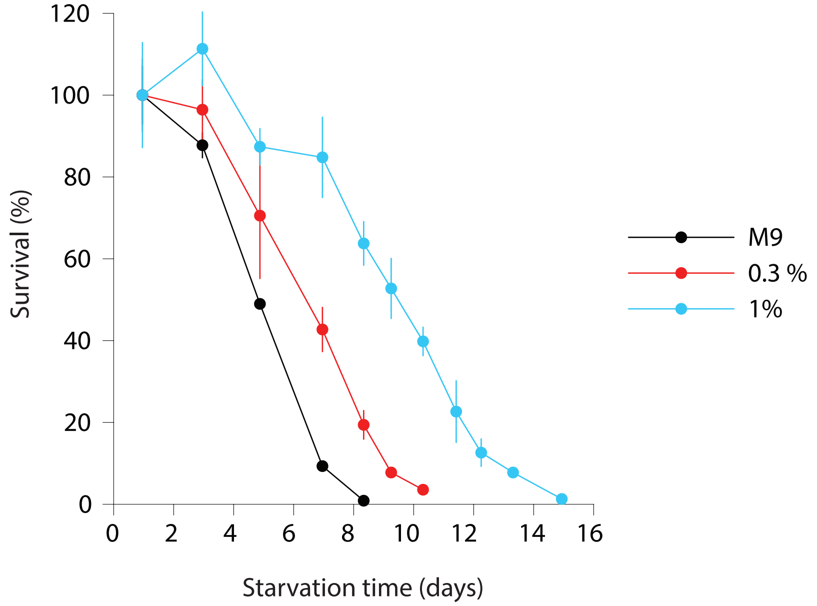

Figure S11. Effect of D-glucose on *daf-6* (CB1377) L1 starvation survival.

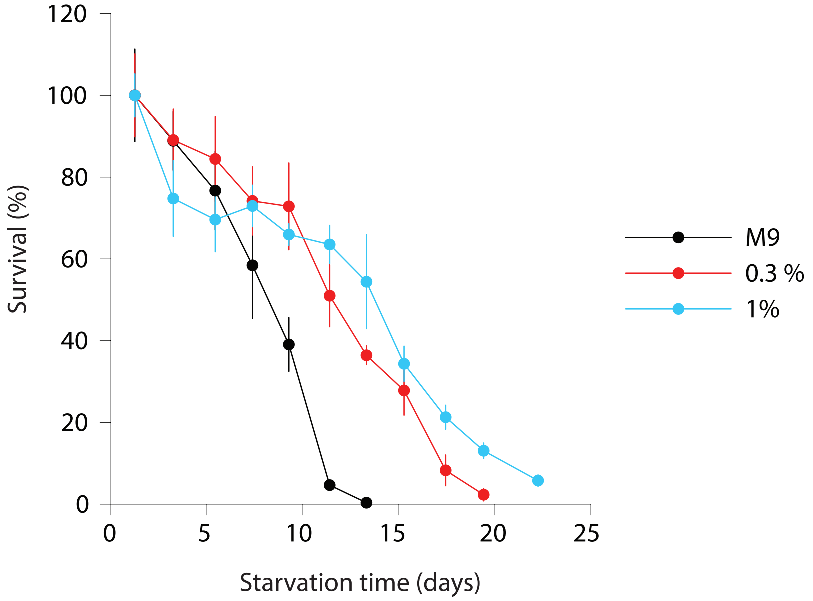

Figure S12. Effect of D-glucose on *osm-1* (PR808) L1 starvation survival.

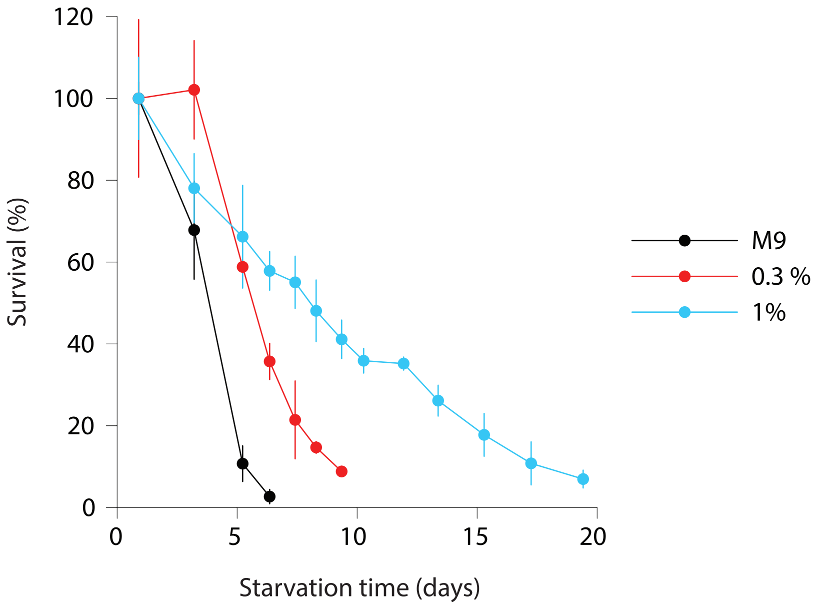

Figure S13. Effect of D-glucose on *C. briggsae* (AF16) L1 starvation survival.

a

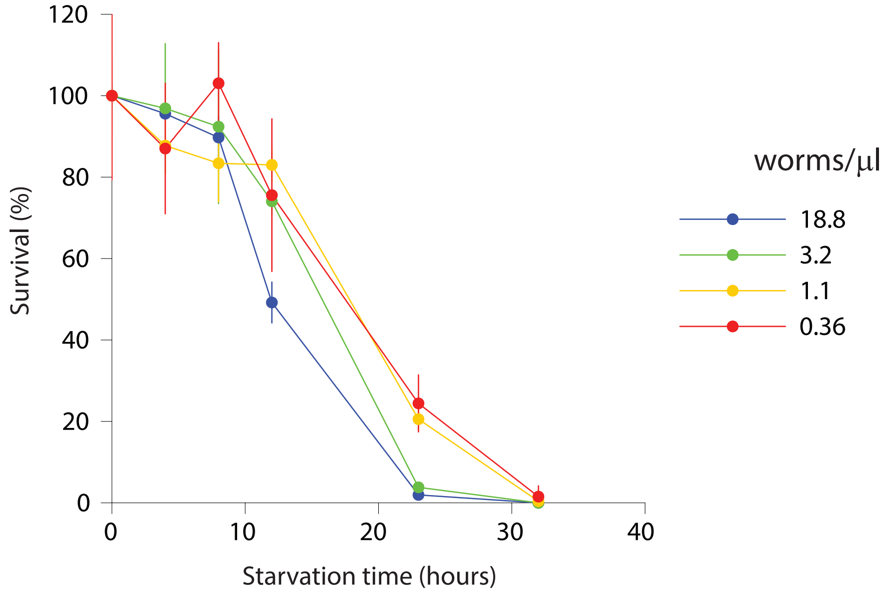

b

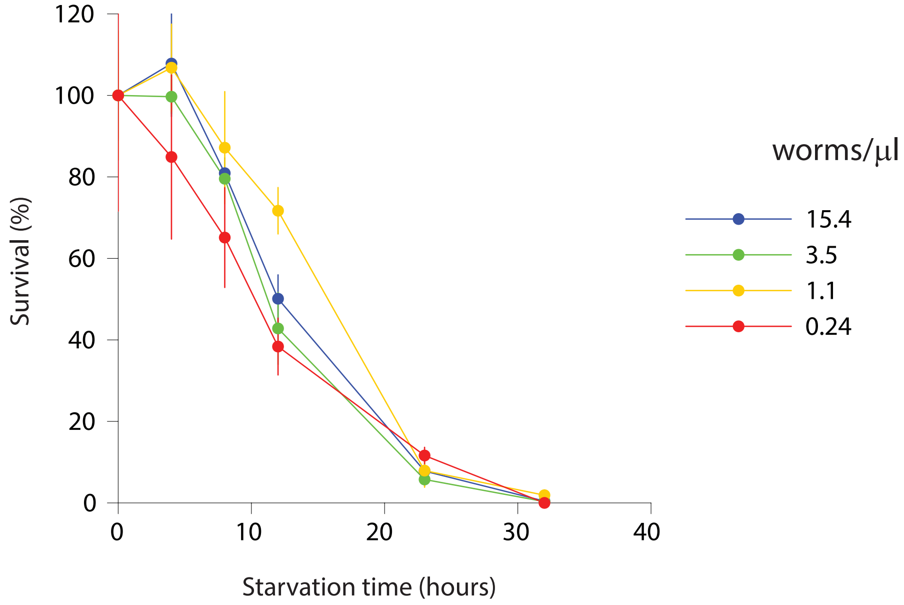

Figure S14. Survival curves of two *C. briggsae* wild isolates in L1 starvation at 34^{0}C at different worm densities, (a) AF16, (b) HK104.

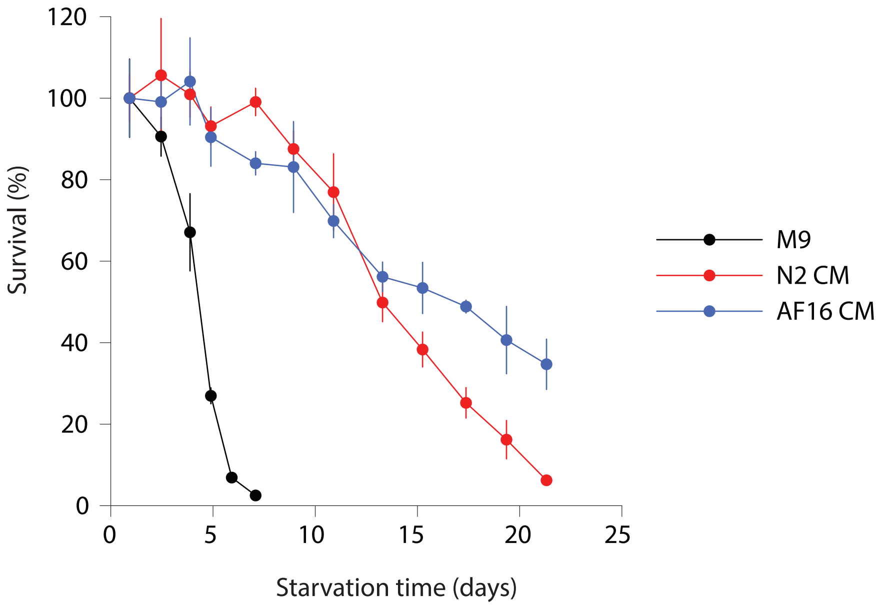

Figure S15. CM600 from *C. elegans* (N2) and *C. briggsae* (AF16) extended AF16 L1 starvation survival.

Table S1. Results of statistical analysis.

Testing significance that…	***Figure***	***Slope****	***SE****	***N****	***p-value***
Slope of *C. elegans* (N2) density dependence is different from zero	1a, 1c, 1d	0.81	0.08	7	$2{\cdot}10^{-4}$
Slope of *C. briggsae* (JU757) density dependence is different from zero	1b, 1c, 1d, 1e	0.07	0.04	6	0.14
Slope of *C. elegans* (JU258) density dependence is different from zero	1c, 1e	0.63	0.06	4	$9{\cdot}10^{-3}$
Slope of *C. elegans* (CB4856) density dependence is different from zero	1c, 1e	0.8	0.2	4	$5.9{\cdot}10^{-2}$
Slope of *C. elegans* (ED3077) density dependence is different from zero	1c, 1e	0.71	0.08	4	$1.4{\cdot}10^{-2}$
Slope of *C. elegans* (ED3072) density dependence is different from zero	1c, 1e	0.9	0.1	4	$2.2{\cdot}10^{-2}$
Slope of *C. briggsae* (HK104) density dependence is different from zero	1c, 1e	-0.08	0.04	6	0.13
Slope of *C. briggsae* (AF16) density dependence is different from zero	1c, 1e	-0.07	0.02	5	$4.5{\cdot}10^{-2}$
Slope of *C. briggsae* (VT847) density dependence is different from zero	1c, 1e	0.02	0.08	4	0.87
Slope of *C. briggsae* (JU439) density dependence is different from zero	1c, 1e	0.02	0.02	5	0.42
Slope of *C. briggsae* (HK104) density dependence is different from slope of *C. elegans* (N2) density dependence	1c				$4{\cdot}10^{-6}$
Slope of *C. briggsae* (JU757) density dependence is different from slope of *C. elegans* (N2) density dependence	1c				$1{\cdot}10^{-5}$
Slope of *C. briggsae* (AF16) density dependence is different from slope of *C. elegans* (N2) density dependence	1c				$5{\cdot}10^{-6}$
Slope of *C. briggsae* (VT847) density dependence is different from slope of *C. elegans* (N2) density dependence	1c				$2{\cdot}10^{-4}$
Slope of *C. briggsae* (JU439) density dependence is different from slope of *C. elegans* (N2) density dependence	1c				$1{\cdot}10^{-5}$
Slope of *C. remanei* (EM464) density dependence is different from zero	1d, 1e	0.99	0.07	5	$6{\cdot}10^{-4}$
Slope of *C. brenneri* (SB280) density dependence is different from zero	1d, 1e	0	0.1	4	0.79
Slope of *C. sp 9* (JU1422) density dependence is different from zero	1d, 1e	0.03	0.06	5	0.63
Slope of *C. sp 3* (PS1010) density dependence is different from zero	1d, 1e	-0.01	0.01	4	0.74

Slope of *C. sp 7* (JU1199) density dependence is different from zero	1d, 1e	0.04	0.06	4	0.61
Slope of *C. japonica* (DF5081) density dependence is different from zero	1d, 1e	-0.04	0.02	4	0.19
Slope of *C. sp 6* (EG4788) density dependence is different from zero	1e	0.79	0.08	4	$1.0 \cdot 10^{-2}$
Slope of *C. sp 11* (JU1373) density dependence is different from zero	1e	-0.2	0.1	4	0.31
Slope of *C. sp 10* (JU1333) density dependence is different from zero	1e	0.44	0.07	4	$2.5 \cdot 10^{-2}$
N2: 50% survival time in M9 is different from 50% survival time in N2 CM60	2a				$<10^{-6}$
N2: 50% survival time in M9 is different from 50% survival time in N2 CM200	2a				$<10^{-6}$
N2: 50% survival time in M9 is different from 50% survival time in *daf-22* CM63.5	2b				$9 \cdot 10^{-5}$
N2: 50% survival time in M9 is different from 50% survival time in *daf-22* CM500	2b				$<10^{-6}$
N2: 50% survival time in M9 is different from 50% survival time in N2 CM60	2c				$<10^{-6}$
N2: 50% survival time in M9 is different from 50% survival time in AF16 CM60	2c				$1 \cdot 10^{-6}$
AF16: 50% survival time in M9 is different from 50% survival time in N2 CM60	2d				$1.6 \cdot 10^{-2}$
AF16: 50% survival time in M9 is different from 50% survival time in AF16 CM60	2d				0.25
Slope of *tax-4* (PR678) density dependence is different from zero	3	0.23	0.05	4	$3.9 \cdot 10^{-2}$
Slope of *osm-6* (PR811) density dependence is different from zero	3	0.20	0.05	5	$2.1 \cdot 10^{-2}$
Slope of *osm-3* (PR802) density dependence is different from zero	3	0.26	0.06	4	$4.4 \cdot 10^{-2}$
Slope of *che-2* (CB1033) density dependence is different from zero	3	0.13	0.02	4	$3.4 \cdot 10^{-2}$
Slope of *daf-6* (CB1377) density dependence is different from zero	3	0.14	0.01	4	$7 \cdot 10^{-3}$
Slope of *osm-3* (MT3762) density dependence is different from zero	3	0.35	0.03	4	$6 \cdot 10^{-3}$
Slope of *osm-1* (PR808) density dependence is different from zero	3	0.21	0.03	4	$2.7 \cdot 10^{-2}$
Slope of *odr-3* (CX2205) density dependence is different from zero	3	0.5	0.1	4	$3.2 \cdot 10^{-2}$
Slope of *ttx-1* (PR767) density dependence is different from zero	3	0.5	0.1	4	$7.0 \cdot 10^{-2}$
Slope of PR679 density dependence is different from zero	3	0.69	0.03	4	$2 \cdot 10^{-3}$

N2: 50% survival time at high density (control) is different from 50% survival time at high density (washed)	4		$7.0\cdot10^{-2}$
N2: 50% survival time at low density (control) is different from 50% survival time at high density shifted to low density	4		0.57
N2: 50% survival time at high density (control) is different from 50% survival time at high density shifted to low density	4		$<10^{-6}$
N2: 50% survival time in M9 is different from 50% survival time in CM	5		$<10^{-6}$
N2: 50% survival time in M9 is different from 50% survival time in combination of fractions	5		$<10^{-6}$
N2: 50% survival time in M9 is different from 50% survival time in FT+WR fraction	5		$1.3\cdot10^{-2}$
N2: 50% survival time in M9 is different from 50% survival time in 50% MeOH fraction	5		$6.7\cdot10^{-5}$
N2: 50% survival time in M9 is different from 50% survival time in MeOH fraction	5		$2.8\cdot10^{-4}$
N2: 50% survival time in M9 is different from 50% survival time in *i*-PrOH fraction	5		$3.3\cdot10^{-2}$

**Slope* of the density dependence was obtained from a linear regression of 50% survival times as a function of $\log_2$(worm density). *SE* is the standard error of the mean *Slope*. *N* is the number of data points for the linear regression.

Supplemental References

S1. Kiontke, K. C., Felix, M. A., Ailion, M., Rockman, M. V., Braendle, C., Penigault, J. B. & Fitch, D. H. A phylogeny and molecular barcodes for Caenorhabditis, with numerous new species from rotting fruits. *BMC Evol. Biol.* **11**, 339 (2011).

S2. von Reuss, S. H., Bose, N., Srinivasan, J., Yim, J. J., Judkins, J. C., Sternberg, P. W. & Schroeder, F. C. Comparative metabolomics reveals biogenesis of ascarosides, a modular library of small-mole cule signals in C. elegans. *J. Am. Chem. Soc.* **134**,1817-1824 (2012).

S3. Lu, N. C. & Goetsch, K. M. Carbohydrate Requirement of Caenorhabditis-Elegans and the Final Development of a Chemically-Defined Medium. *Nematologica* **39**, 303-311 (1993).
